# GOMMA: a component-based infrastructure for managing and analyzing life science ontologies and their evolution

**DOI:** 10.1186/2041-1480-2-6

**Published:** 2011-09-13

**Authors:** Toralf Kirsten, Anika Gross, Michael Hartung, Erhard Rahm

**Affiliations:** 1 Interdisciplinary Centre for Bioinformatics, Universität Leipzig, Härtelstraße 16-18, 04107 Leipzig, Germany; 2 LIFE - Leipzig Research Centre for Civilization Diseases, Universität Leipzig, Philipp-Rosenthal-Straße 27, 04103 Leipzig, Germany; 3 Department of Computer Science, Universität Leipzig, Johannisgasse 26, 04103 Leipzig, Germany

## Abstract

**Background:**

Ontologies are increasingly used to structure and semantically describe entities of domains, such as genes and proteins in life sciences. Their increasing size and the high frequency of updates resulting in a large set of ontology versions necessitates efficient management and analysis of this data.

**Results:**

We present GOMMA, a generic infrastructure for managing and analyzing life science ontologies and their evolution. GOMMA utilizes a generic repository to uniformly and efficiently manage ontology versions and different kinds of mappings. Furthermore, it provides components for ontology matching, and determining evolutionary ontology changes. These components are used by analysis tools, such as the Ontology Evolution Explorer (OnEX) and the detection of unstable ontology regions. We introduce the component-based infrastructure and show analysis results for selected components and life science applications. GOMMA is available at http://dbs.uni-leipzig.de/GOMMA.

**Conclusions:**

GOMMA provides a comprehensive and scalable infrastructure to manage large life science ontologies and analyze their evolution. Key functions include a generic storage of ontology versions and mappings, support for ontology matching and determining ontology changes. The supported features for analyzing ontology changes are helpful to assess their impact on ontology-dependent applications such as for term enrichment. GOMMA complements OnEX by providing functionalities to manage various versions of mappings between two ontologies and allows combining different match approaches.

## Background

Ontologies and taxonomies have become increasingly important especially in the life sciences [1,2]. They are predominantly utilized to structure and uniformly describe the entities of a domain of interest such as molecular functions or the anatomy of species [3,4]. Ontologies consist of a set of concepts that are usually interrelated by "*is-a*", "*part-of*" or other semantically meaningful relationships (e.g., "*regulated-by*") [5]. Ontologies enable a consistent annotation of biological objects, experiments, publications or clinical documents by describing their properties. For instance, the Molecular Function ontology of the Gene Ontology [6] is used to specify the functions of genes and proteins on the molecular level. Biomedical ontologies are typically provided in different formats including Web Ontology Language (OWL) and the Open Biomedical Ontologies (OBO) Flat File Format.

There exist several infrastructures providing access to life science ontologies such as the OBO Foundry [7], Ontology Lookup Service (OLS) [8] and BioPortal [9]. Table 1 summarizes features of these (and other) platforms that will be discussed later in the Discussion section. In this paper, we propose a new infrastructure called GOMMA (**G**eneric **O**ntology **M**atching and Mapping **Ma**nagement) augmenting the existing systems with additional functionality. In particular, GOMMA analyzes the change history or evolution of ontologies and provides advanced functionality to help ontology users to better deal with ontology changes. The need for such functionality follows from the observation that life science ontologies undergo continuous modifications to better reflect new research results and community agreements. Typically, there are several new versions per ontology and year; new versions of the heavily used Gene Ontology are even released on a daily basis. Ontology modifications may invalidate annotations [10] and influence applications such as ontology-based functional profiling of gene sets [11,12]. GOMMA includes algorithms to automatically detect the changes between ontology versions and, can thus, help to identify, study and resolve problems caused by such changes.

**Table 1:** Comparison of existing platforms and systems that provide and apply life science ontologies

	OLS	OBO	BioPortal	GOMMA	SAMBO
**Description**	Service to query, browse and navigate biomedical ontologies	Collaborative platform having shared principles to govern and coordinate ontology development	System to access and share ontologies that are actively used in biomedical communities	Infrastructure to manage, analyze and match ontologies taking their evolution into account	System for aligning and merging biomedical ontologies

**Supported ontology formats**	OBO	OBO, OWL	OBO, OWL, RDF, RRF, ...	OBO, OWL, RDF, ... (extensible via flexible importers)	OWL

**Ontology mappings**	-	x	x	x	x

**Versioning support**	- (only latest versions are accessible)	x (downloadable versions via CVS repository)	x (access and download of ontology versions)	x (efficient versioning of ontologies in a repository)	- (no explicit ontology versioning possible)

**Evolution mappings**					
**1) change log**	-	x (information about changes via newsletters)	-	- (version comparison to detect changes)	-
**2) complex diff**	-	-	-	x	-

**Match support/functionality**	-	-	-	x (metadata, external knowledge, instances)	x (metadata, external knowledge, documents, learner)

**Search, navigation**	ontologies	ontologies	ontologies and mappings	ontologies, ontology evolution	-

**"Special Functionalities"**	auto completion, term hierarchies via graphs	discussion lists and wiki to support collaborative development	automatic text annotation	enhanced Diff, annotation migration	merging, user interaction

**Availability and Access**					
**1) download (ontologies, mappings)**	x	x	x	-	-
**2) visualization/GUI**	x (web application))	- (infrastructure to share the ontologies)	x (web portal)	x (web application OnEX)	x (desktop GUI)
**3) API/web service**	x (web service: Ontology QueryService	-	x (web service to access and query available on tologies)	x (query ontology and mapping versions, statistics, diff, match via API)	unknown

The table provides a comparative overview of platforms that provide and apply life science ontologies. The systems are compared by different characteristics such as versioning support or search/navigation facilities.

**Figure 1:**
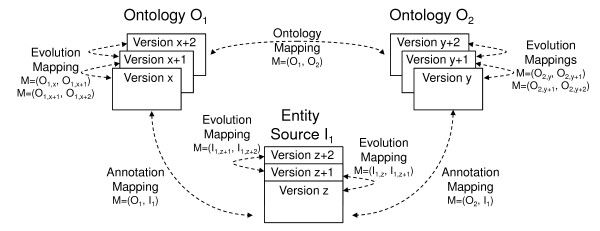
**Versions of ontologies and entity sources and mappings among them**. The figure shows the versioning of ontologies and entity sources and their interrelation using ontology, annotation, and evolution mappings.

Furthermore, GOMMA supports the management of different kinds of ontology-based mappings describing how ontologies are related to other ontologies or how they relate to biomedical entities such as gene or protein descriptions. Figure 1 illustrates how *ontologies*, entity *sources *as well as their versions are related in the life science domain leading to different types of *mappings *among them. Entity sources like the genome source Ensembl [13] or Swiss-Prot [14] use ontology concepts to uniformly describe or annotate their objects (e.g., genes). Such a set of links between an ontology and entity source forms a so-called *annotation mapping*. Moreover, *ontology mappings *contain correspondences between overlapping ontologies that interrelate semantically equivalent or related ontology concepts. Changes to ontologies and entity sources are reflected in regularly released new versions; changes between two succeeding versions can be captured by so-called *evolution mappings*. Changes of ontologies and entity sources may make it necessary to adapt the dependent ontology and annotation mappings accordingly. GOMMA helps to address these problems by determining evolutionary changes of ontologies and their mappings as well as supporting different kinds of evolution analysis.

While entity and annotation mappings are usually provided by the source providers, ontology mappings typically need to be explicitly identified. Such mappings are valuable for overlapping ontologies describing objects of the same domain, e.g., the human anatomy. The semantic correspondences of ontology mappings can be used for many tasks, e.g., to find new annotations, to combine (merge) related ontologies or to support other data integration scenarios [15,16]. As many of today's ontologies are large with up to thousands of concepts, a manual determination of ontology mappings is often infeasible. Therefore, a semi-automatic detection of correspondences by *ontology matching *methods becomes necessary. The GOMMA infrastructure supports different match techniques to create ontology mappings as well as to align different versions of an ontology to determine evolutionary changes.

In this paper we introduce the GOMMA infrastructure and discuss some of its tools/applications. In particular, we make the following contributions:

• We describe the component-based infrastructure GOMMA to manage, match, and analyze many versions of different life science ontologies. GOMMA is organized in a modular manner and can be flexibly adapted and extended. A generic repository manages the versions of ontologies and entities of interest as well as the different kinds of mappings. The GOMMA infrastructure is distributed such that tasks like ontology matching can be executed in parallel on several computing nodes to reduce execution time and memory requirements. The GOMMA prototype is provided on our website at http://dbs.uni-leipzig.de/GOMMA.

• We briefly describe GOMMA-based functions for the life science community such as the online evolution analysis tool OnEX [17], the COntoDiff approach for determining complex ontology changes [18], and the Region Analyzer to detect stable and unstable ontology regions [19].

• We describe a typical life science application affected by ontology changes and thus requiring a continuous evolution analysis. In particular, we illustrate the impact of ontology changes on analysis results for ontology-based term enrichment.

The main focus of this paper is on the GOMMA infrastructure and its methods for managing ontology versions and mappings as well as for analyzing the evolution of ontologies. The Methods section outlines the main methods of GOMMA. In the Results section, we illustrate the use of GOMMA functionality for analyzing the evolution of life science ontologies in a typical application scenario.

## Methods

We start with an overview of the GOMMA infrastructure and then describe specific components and methods.

### Overview of the GOMMA Infrastructure

**Figure 2:**
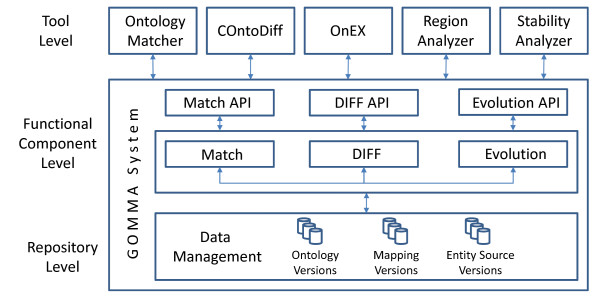
**Overview of GOMMA's component-based infrastructure**.

Figure 2 shows the architecture of the component-based GOMMA infrastructure. It consists of three levels, namely *repository*, *functional components*, and *tools*. The repository centrally and uniformly manages all versions of ontologies, entities, and the different kinds of mappings (annotation mappings, ontology mappings, and evolution mappings) introduced in the Background section (see also Figure 1).

The managed ontologies, entities and mappings are used by three main functional components called *Match*, *DIFF *and *Evolution*. The Match component is used to determine an ontology mapping between two ontologies by calculating the semantic similarity between their elements. For this purpose, this component provides various similarity and distance functions taking ontology metadata, associated entities or both into account. The DIFF component is responsible for determining an evolution mapping between succeeding versions of an ontology or entity source. It includes several functions to detect basic changes such as element additions and deletions as well as complex changes such as merging multiple concepts into one concept. Computed ontology and evolution mappings can be stored in the repository. Finally, the evolution component supports evolution analysis taking the history of ontologies and entity sources into account.

There are functional dependencies between these three functional components. The Match component can determine mappings between two ontology versions which are then used by the DIFF component to find changed ontology portions. Additionally, evolution mappings determined by the DIFF component are utilized by the Evolution component to create statistics and analyze the change history. These functional components use a central component to access ontology versions, entity sources and mapping data managed in the repository. This repository access component also enables the import of additional source versions and mappings. All functionalities are accessible by component-specific APIs.

The top layer in Figure 2 consists of tools utilizing the GOMMA infrastructure and its functionality. The *Ontology Matcher *primarily uses the Match component to determine semantic relationships between two ontologies whereas *Complex Ontology Diff *(COntoDiff) can recognize basic as well as complex change operations between different ontology versions. Ontology changes can be explored and visualized by the *Ontology Evolution Explorer *(OnEX) tool. The *Region Analyzer *permits evaluating which regions within ontologies are highly changed or stable primarily within a time period of interest.

In the following, we describe the methodology of selected components in more detail. We start with the versioning concept used in GOMMA, especially for managing data within the repository. We then describe the functional components.

### Uniform Representation of Ontologies, Entities, and Mappings

The basis of the GOMMA infrastructure is the repository. It uniformly manages all versions of ontologies, entity sources and mappings using a generic graph-based structure. Each ontology (entity source) *S *= (*E*, *R*, *A*) is represented by a set of elements *E*, such as ontology concepts and entities, which are interrelated by a set of relationships *R*: *E *× *E*. Each *r *= (*r_Source_, r_Type_, r_Target_*) of *R *connects the elements *r_Source _*and *r_Target _*by a relationship type *r_Type_*. Elements are described by a set of attributes *A*, e.g., concept name and description or source-specific attributes for entities. All elements are assumed to support a unique identifier or *accession number *attribute. A mapping *M *= (*S_1_*, *S_2_*, *C*, *T*, *P*) between two sources (ontologies) *S_1 _*and *S_2 _*consists of a set of correspondences *C *associating elements of *S_1 _*with those of *S_2_*. Each correspondence *c *= (*s_1_, s_2_, type*) is associated with a semantic type, e.g., equivalence and parthood. The mapping *M *is of a specific type *T *(annotation or ontology mapping) and can be described by a further set of describing properties *P *including the mapping source, the utilized computing tool, or the name of the person who created the mapping or additional mapping classifications and types, respectively.

A version *v *of source *S *is denoted with version *S_v _*= (*E*, *R*, *A*, *t*) and reflects the state of source *S *at a specific point in time *t*. GOMMA manages different versions of sources and mappings. It utilizes the observation, that versioning is typically linear, i.e., for each source (ontology) version *S_i _*there exists at most one preceding version S_i-1 _and one succeeding version *S_i+1 _*such that there is a chain of succeeding source versions *S_0 _*..., *S_i-1_*, *S_i_*, *S_i+1_*,... *S_n_*. Therefore, a source version including all its elements is created at a specific point in time and continuously exists unless its maintenance stops at some point in the future. In general, GOMMA stores source elements, i.e., ontology concepts and entities, only once and maintains their lifetime [20]. The lifetime is represented by a start date *t_Start _*and an end date *t_End_*. Since every version is associated with a version date *t*, GOMMA is able to rebuild any source version at query runtime by selecting all elements for that hold *t_Start _*≤ *t *<*t_End_*. The lifetime-based versioning implementation is also utilized for relationships interrelating the elements of a source.

Typically, the element representations of different ontologies and entity sources are very heterogeneous, i.e., ontology concepts and entities are described by a large variety of attributes. While ontologies often utilize attributes like *name*, *definition*, *description *and perhaps *synonyms*, the attributes of entity sources are usually very specific. For instance, the genome source Ensembl [13] uses attributes *chromosome*, *start *and *stop positions *and *strand *to describe the localization of genes, transcripts and translations. Since the values of attributes frequently change over time it is necessary to capture attribute value changes within different ontology and entity source versions as well. Hence, a flexible repository schema including versioning support is required to uniformly manage the diverse attributes and their values on the one hand and to efficiently manage different versions of them on the other hand. GOMMA does not include the ontology and entity source attributes in its schema but utilizes a *generic attribute-value concept *for improved flexibility [21]. Furthermore, the versioning concept of elements is also applied for attribute value combinations. In particular, they have an associated lifetime making it possible to maintain a history of attribute values.

The repository with the proposed versioning concept has been implemented with the relational database system MySQL providing us with the SQL query language to retrieve and modify repository data. In [20] we evaluated this implementation according to runtime and space efficiency by comparing the versioning approach with the naive versioning method in which each version is stored separately. As a result, we observed that our versioning approach significantly reduces the space requirements the more versions need to be managed.

The generic internal structure of GOMMA allows to import and manage many ontologies of different formats. Various import functions utilizing public archives of ontology distributors including the CVS repositories of the OBO ontologies and the Gene Ontology archive transform different ontology representations into the internal repository structure. Different ontology formats, such as OBO and OWL, can be converted to the repository structure by aligning OBO terms/OWL classes with GOMMA elements. The OBO relationships and OWL properties and axioms are represented by GOMMA relationships. For instance, *subClassOf *relations in OWL and *is_a *relationships in OBO are captured by the relationship type *is-a *in GOMMA. Other more biomedical-specific types, such as *regulates *or *is-regulated-by*, are represented by equivalent relationship types in GOMMA. A broader and more theoretical founded discussion of commonalities and differences between different ontology representations including semantic networks, conceptual graphs and description logics is given in [22].

### A Component for Matching Ontologies

GOMMA provides comprehensive support for ontology matching to semantically align two given life science ontologies *O *and *O'*. The match result is an ontology mapping consisting of correspondences, i.e., pairs of semantically equivalent or related concepts of the input ontologies. We use a match operation *MO *= (*O*, *O'*, *A*, *K*) to compute a mapping between ontologies *O *and *O' *based on an alignment (match) method *A *and optionally further knowledge *K*, e.g., thesauri, associated entities, or further background knowledge. There is a large number of proposed match algorithms making use of numerous similarity and distance functions to quantify the semantic relatedness of ontology concepts (see [23-28] for overviews). The approaches can be classified into metadata-, annotation-based and hybrid approaches. Metadata-based match approaches utilize ontology metadata for alignment, such as concepts names, definitions but also the ontology structure. By contrast, annotation-based approaches evaluate the entities associated to ontology concepts. The key idea behind these approaches is that two concepts are semantically related if they share a significant number of entities or if they have highly similar entities. Hybrid match approaches combine metadata- and annotation-based approaches. In general, match methods can only determine candidate correspondences that need to be verified and corrected by human experts. Furthermore, some correspondences may not be found by automatic methods. To improve the computed ontology mappings and thus to reduce the manual effort for correcting them, it is generally not sufficient to rely on a single match method but one has to combine several matchers that may result in complex match workflows. Computing such complex workflows even for large input ontologies is often a resource and time intensive process requiring special performance optimizations such as pruning and parallel ontology matching [25]. GOMMA supports the parallel execution of different (independent) matchers on the same input ontologies, but also the internal parallelization of individual matchers based on partitioning of input ontologies [29].

**Figure 3:**
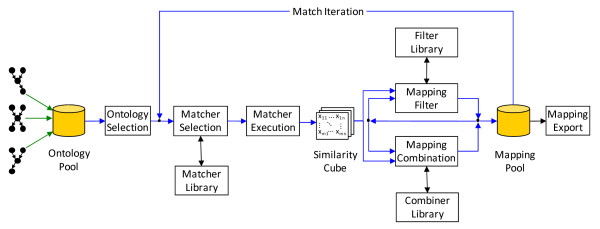
**The match process in GOMMA**. GOMMA utilizes the sketched process to create ontology mappings. This process iteratively generates mappings between selected input ontologies and includes feedback from human experts.

Figure 3 shows a typical match process performed in GOMMA; the approach is inspired by the ontology matching system COMA++ [30] supporting the combined application of several matchers (match algorithms). The ontologies to be matched have at first to be integrated into the GOMMA repository. Match processing for two ontologies then entails the execution of several matchers that are selected from the library of supported matchers. Each matcher generates an intermediate mapping result represented by a matrix of computed similarity values (0 ≤ similarity ≤ 1) for pairs of concepts from the input ontologies. The similarity matrices are aggregated into a single similarity matrix (according to some selected combination strategy) in order to obtain a combined (still intermediate) match result. Furthermore, a filter step is applied (according to some selected filter strategy) to select the most likely correspondences for the mapping result. The determined ontology mapping can be stored within a mapping pool in the GOMMA repository. The match process may be applied iteratively to refine and improve an initially generated ontology mapping. Additionally, human experts can provide feedback to verify and correct computed mappings.

GOMMA includes several match-related libraries providing a large number of metadata- and annotation-based matchers as well as approaches for mapping combination (aggregation) and filtering. This approach also facilitates easy extensibility to support additional match approaches. Table 2 lists the current metadata-based and annotation-based matchers of GOMMA. The metadata-based matchers include a linguistic matcher that determines the similarity of two concepts based on the linguistic similarity of the concept names or their synonyms. The linguistic similarity, in turn, may be determined according to different approximate string similarity measures such as Levenshtein or N-gram. The path matcher is a simple structural matcher that considers all parent concepts up to the ontology root for determining the similarity between concepts. GOMMA's annotation-based matchers assume the existence of annotation mappings between the input ontologies and an entity source, e.g., GO annotations for proteins [31] or MeSH annotations of PubMed publications [32]. Using these annotation mappings, GOMMA aligns two ontologies by evaluating to what degree the entities of two concepts overlap. This overlap is translated into concept similarities according to different functions, such as Dice, Jaccard, and Cosine (see [33-36] for details and example studies).

**Table 2:** Selected matchers of GOMMA

Category	Name	Description
*Metadata-based* *Matcher*	Linguistic Matcher	This matcher computes the linguistic similarity between two ontology concepts. The matcher is configured by two sets of attributes specifying which attribute values are used to align the concepts of O and O'. The linguistic similarity functions include nGram, Loom, and others.
	Child Matcher	The child matcher computes the similarity between two ontology concepts based on the similarity of their children.
	Path Matcher	The path matcher computes the similarity between two ontology concepts taking the paths from the concepts to their root element into account. Each path is represented by concatenating concept names. Finally, the matcher computes the linguistic similarity between the paths.
	Similarity Flooding	This structural matcher computes the similarity between two concepts based on the Similarity Flooding algorithm.
*Annotation-based Matcher*	Annotation-based Concept Matcher	The annotation-based matcher computes the similarity between two ontology concepts by taking the associated entities into account. The matcher utilizes an annotation mapping to determine the degree of shared entities of two concepts to compare. The similarity functions include Dice, Jaccard, and Cosine.

The table lists selected metadata-based and annotation-based matchers of GOMMA. The matchers take two ontologies O and O' as input and produce a set of correspondences interrelating the input ontologies. All matchers are configured by the similarity function that is used to compute the concept similarity of correspondences.

GOMMA does not keep all intermediate correspondences but filters out early those correspondences whose similarity (confidence) is very low to limit the memory requirements for matching. Multiple ontology mappings resulting from the application of different matchers are combined by typical set operations like union, intersection and difference but also by other approaches such as majority voting where a correspondence is accepted if it is determined by the majority of matchers. These combination operations are configured with a specific aggregation function, such as maximum, minimum, and average, to derive a combined confidence value from the matchers' individual confidence values. GOMMA also provides multiple filters to finally select the correspondences from the aggregated mapping. Simple filters like *ConfidenceThreshold *only keep correspondences with a confidence higher than the specified threshold. More sophisticated filters consider structural mapping properties such as support for "stable marriages" where a correspondence between concepts c_1 _and c_2 _is only accepted if c_2 _is the most similar element for c_1 _and vice versa.

The Match component can be used in multiple ways. First, it provides several methods to interrelate knowledge covered by different ontologies. GOMMA supports similar linguistic matchers than [37,38] as well as a scalable match approach based on the composition of existing ontology mappings. The evaluation in [39] showed that composing mappings for large ontologies, e.g. UMLS [40], is highly effective in terms of mapping quality. The Match component can also be used to align two versions of the same ontology to determine which elements are unchanged and which ones are new or missing in the new version.

### Detecting Changes among Ontology Versions

Usually, ontology providers regularly release updated versions of their ontologies to reflect the latest research insights or community agreements. Typically, the changes are informally discussed on mailing lists and, thus, cannot be automatically processed in a generic manner for all ontologies. This makes it difficult for users of ontologies to determine whether their applications or mappings are affected by recent ontology changes, e.g., if annotation mappings need to be adapted due to deletions or changes of previously used ontology concepts.

The DIFF component of GOMMA implements several algorithms to detect changes between two versions of an ontology. In line with the GOMMA versioning concept, we assume linear sequences of ontology versions *O_0_*, ... , *O_i-1_*, *O_i_*, *O_i+1_*, ... , *O_n_*. An ontology version *O_v _*= (*C*, *R*, *A*, *t*) reflects the state of ontology *O *at a specific point in time *t*. It consists of a set of concepts *C*, a set of relationships *R*: *C *× *C *and a set of concept attributes *A*. Changes between two ontology versions *O_i _*and *O_j _*are captured in an *evolution mapping diff*(*O_i_*, *O_j_*, *Changes*) which highlights the version differences. The DIFF component distinguishes two types of changes (and thus two types of evolution mappings): (1) *basic changes *and (2) *complex changes*. Basic changes comprise the simplest ontology modifications namely *add *and *delete *which can be applied to concepts, relationships and attributes. The addition and deletion of concepts and relationships can be easily detected by comparing two ontology versions *O_i _*and *O_j _*(*i *<*j*) taking the concept identifier (accession number) into account. Attribute changes, i.e. the modification of attribute values such as concept name or description, represent a further kind of basic change. In the result section, we introduce the web application OnEX for analyzing basic changes.

Complex changes are based on basic changes or other complex changes and thus specify changes at a higher level of abstraction. Examples of such complex change operations include the *split *and *merge *(*fuse*) of concepts or the addition/deletion of entire ontology regions (*addSubGraph*, *delSubGraph*). The split operation is applied when two or more concepts are newly introduced in the new version *O_j _*and replace a single concept of the old version *O_i_*. By contrast, the merge operation fuses two or more concepts of *O_i _*to a single concept in *O_j_*. We apply a rule-based change detection [18] to identify such complex changes. The approach first performs a match operation between the two ontology versions *O_i _*and *O_j _*to determine the corresponding ontology portions and then applies so-called Change Operation Generating (COG) rules to iteratively derive the basic as well as complex changes that took effect between two ontology versions. In the Results section, we present results of the COntoDiff tool which is used to determine complex, i.e., expressive and semantically rich changes between two ontology versions.

While the DIFF change detection algorithms can be executed on demand, GOMMA already determines basic changes whenever a new ontology version is imported. In particular, it compares the imported version and its predecessor version if available within the repository by applying the change detection algorithms. The analysis results are materialized in the repository. Hence, the results can be used in different applications and analysis scenarios, such as descriptive and frequency statistics but also difference and evolution analysis, without recalculating the change detection whenever the data is needed.

### Evolution Component

GOMMA's change detection is the basis for different kinds of evolution analysis aiming at finding evolution patterns for ontologies and also for entity sources. Such evolution patterns can be used to differentiate between rather stable and heavily changed ontologies, e.g., recently developed ontologies for domains of high research interest. Evolution patterns can also be utilized to find interesting regions within a single ontology which, again, are rather stable or under heavy development. We briefly describe both analysis approaches in the following.

To determine the change activity per ontology we can use basic change frequencies, in particular the absolute number of added, deleted and changed concepts and relationships across different versions [41]. These measures can be normalized according to the total number of concepts and relationships, respectively. Additionally, ratios, such as the *add-delete-ratio *(number of added vs. deleted ontology concepts between two ontology versions) can reveal relevant change patterns. Relative measures and ratios are better suited than absolute change rates to compare the change intensity between different succeeding versions of an ontology as well as among different ontologies at a specific time. In the Results section, we show selected evaluation and analysis results.

For a specific ontology of interest, we can further determine ontology regions that are under heavy development or, conversely, are rather stable. Such regions are of potential interest for domain researchers as well as ontology curators. For instance, domain researchers may be interested in evolving areas, or the information about new ontology regions may be useful for curators to establish new functional annotations. In [19] we describe a method to detect stable and heavily changing ontology regions that allows weighting the costs of different change operations such as deletions and additions. Such change costs are not only determined for ontology concepts but aggregated within connected ontology sub-graphs or regions. The resulting costs can be normalized, e.g., by the region size (number of concepts in the sub-graph) to determine the overall stability of concept regions. The higher the change costs per concept the higher the instability of the region. In contrast, regions with zero change costs are the most stable ones of an ontology. The application of the Region Analyzer allows for the determination of such interesting ontology region (see Results section).

## Results

In this section we describe some of GOMMA's functionality for evolution analysis and its use for a typical ontology-dependent life science application scenario. The relevant GOMMA functionality includes the OnEX web application, the Region Analyzer and COntoDiff. For each function, we present analysis results for different ontologies and show its usability for our example scenario.

### Application scenario: term enrichment analysis

A typical application of life science ontologies is term enrichment analysis or functional profiling of large gene sets of interest. Term enrichment algorithms [11,42-46] use sets of ontology-based annotations to identify significantly over/under-represented categories w.r.t. the considered gene set. This helps to identify significant molecular functions or biological processes (for example) in which the considered genes are commonly involved. Typically, such algorithms propagate functional annotations throughout the ontology and are thus highly dependent on the ontological structure. Hence, the results of such algorithms can be influenced when ontologies evolve over time.

**Figure 4:**
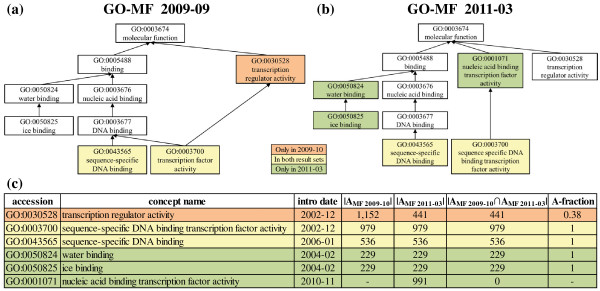
**Application scenario: Term enrichment analysis**. The figure shows analysis results for a term enrichment analysis of a gene set using a hypergeometric test from the FUNC package [11]. The experiment was executed for two Gene Ontology Molecular Function (GO-MF) versions: 2009-09 (a) and 2011-03 (b). The gene and annotation set were not modified. Colored categories denote significantly enriched categories w.r.t. the used gene set and ontology version. The table (c) shows more detailed information for each significant category, e.g., the number of indirect (propagated) gene annotations (|A|).

We run a term enrichment analysis using the hypergeometric test from the FUNC package [11] on a publicly available example data set (http://fasta.bioch.virginia.edu/cshl/stubbs/data/TF1/TF1_ForFUNC_Hyper.txt). This data set was initially based on Gene Ontology version 2009-09. We repeated the analysis using the original as well as a newer GO version (2011-03) and compared the result sets for the Molecular Functions part of GO (GO-MF). Figure 4(a) and 4(b) show the GO-MF subgraphs with significant result categories of this analysis. We observe that the statistical test for the new GO-MF version leads to a significantly changed result set. In particular, one category (in orange color) is no longer in the new result set while three categories (in green) appear as significant only for the new version. Only two categories (yellow) are present in both result sets. This indicates that results of such term enrichment analyses can be highly dependent on ontology evolution and, thus, the used ontology version. When introducing the GOMMA functions for evolution analysis in the following, we also explain their usability for the example scenario.

### OnEX

**Figure 5:**
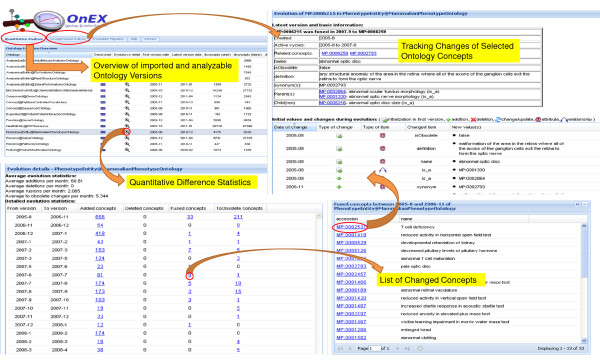
**Evolution statistics in OnEX**. The figure shows selected use cases of the web-based system Ontology Evolution Explorer (http://www.izbi.de/onex). The overview shows statistics for all ontologies currently integrated in OnEX. Tracking changes, the list of changed concepts and quantitative difference statistics are shown for the Mammalian Phenotype Ontology.

The GOMMA-based Ontology Evolution Explorer (OnEX) [17] available at http://www.izbi.de/onex provides change statistics for numerous ontologies and supports interactive exploration of their evolution histories. Currently, OnEX covers more than 780 versions of 16 life science ontologies dating back to 2002. The evolution statistics are available at the level of entire ontologies as well as at the concept and attribute levels. Figure 5 shows the OnEX user interface with exemplary data on the evolution of the Mammalian Phenotype ontology [47]. On the ontology level, details are provided about the first and last available version, the total number of versions, and the number of their concepts and relationships. For a selected ontology, the user can explore how many and which concepts and attributes changed in which way (add, delete, etc.) between succeeding versions (bottom left in Figure 5). Additionally, OnEX allows searching for concepts by specified keywords and lists their changes at the attribute level (right part of Figure 5). Ontology users such as curators or researchers, can thus track all changes in detail, e.g., according to the name, definition or relationships of selected concepts. Common ontology browsers do not provide such detailed information about conceptual and structural ontology changes. Curators may easier review their own changes and thus prepare future revisions, e.g., to correct erroneous changes. For the introduced application scenario, such information may help to explain changes as we will see in the analysis results.

OnEX can also be used to identify and migrate annotations for outdated ontology versions. For this purpose, users provide an annotation mapping of interest and specify to which ontology version it should be migrated. The system reports annotations affected by changed ontology concepts and lets the user decide how to migrate them, e.g., whether an annotation for an obsolete or deleted concept should also be deleted.

Table 3 shows OnEX evolution statistics for 16 selected biomedical ontologies. For each ontology, the number of concepts (*|C|_0_*, *|C|_n_*) and relationships (*|R|_0_*, *|R|_n_*) of the first and the latest version available in GOMMA is listed. Furthermore, simple growth factors (*growth_C_*, *growth_R_*, *growth_C+R_*) are provided specifying the ratio between the number of concepts (relationships, concepts+relationships) in the last vs. the first version. Based on the change intensity, we distinguish three groups of ontologies (separated by strong lines). For instance, the relative growth rates for concepts, *growth_C_*, vary from a factor of almost 5 for the PPI (protein protein interaction) ontology to only 1.08 for the Flybase Controlled Vocabulary. In absolute numbers, NCI Thesaurus and GO Biological Processes have the strongest growth by about 48,000 and ~12,000 concepts, respectively. By contrast, the Flybase Controlled Vocabulary has grown by only ~50 concepts since 2002.

**Table 3:** Evolution statistics for selected biomedical ontologies

Name	O_0_	O_n_	|C_o_|	|C_n_|	growth_C_	|R_0_|	|R_n_|	growth_R_	growth_C+R_
Protein Protein Interaction Ontology	2005-08	2009-06	194	960	4.95	211	1,006	4.77	4.85
Biological Processes (GO)	2002-12	2010-03	6,741	19,099	2.83	0	39,391		8.68
NCI Thesaurus	2003-10	2009-12	28,740	77,448	2.69	33,847	86,803	2.56	2.62
Cellular Components (GO)	2002-12	2010-03	1,124	2,810	2.50	0	5,185		7.11
Chemical Entities of biomedical Interest	2004-10	2009-08	10,236	24,225	2.37	11,592	43,085	3.72	3.08

Mammalian Phenotype Ontology	2005-08	2010-03	4,175	7,571	1.81	4,620	8,560	1.85	1.83
Sequence Ontology	2005-08	2010-03	981	1,764	1.80	1,181	2,014	1.71	1.75
Molecular Functions (GO)	2002-12	2010-03	5,298	9,487	1.79	0	10,972		3.86
Pathway Ontology	2005-11	2010-03	427	751	1.76	478	923	1.93	1.85
Zebrafish Anatomy	2005-11	2009-12	1,389	2,431	1.75	4,272	8,819	2.06	1.99
Cell Type Ontology	2004-06	2010-01	687	1,049	1.53	1,251	1,799	1.44	1.47
Plant Structure Ontology	2005-07	2009-08	681	868	1.27	980	1,274	1.30	1.29
Protein Modification Ontology	2006-06	2009-02	1,074	1,338	1.25	1,568	1,982	1.26	1.26

Adult Mouse Anatomy	2005-08	2010-03	2,416	2,947	1.22	2,939	3,722	1.27	1.25
Fly Anatomy	2004-12	2010-02	6,090	6,707	1.10	9,826	12,319	1.25	1.20
Flybase Controlled Vocabulary	2004-12	2010-02	658	713	1.08	653	698	1.07	1.08

The table shows evolution statistics of analyzed ontologies taking different evolution measures such as the growth rate or the number of concepts/relationships into account. (|R| - number of relationships, |C| - number of concepts)

**Figure 6:**
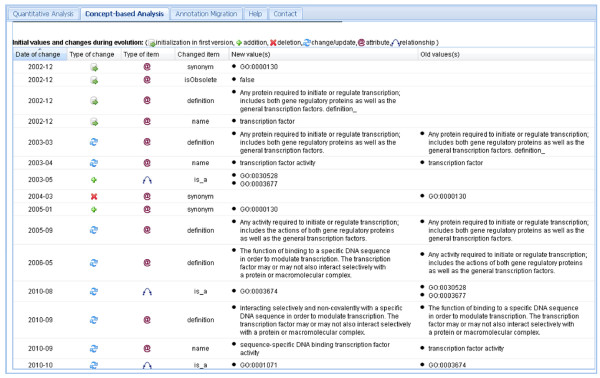
**Change history of GO:0003700 in OnEX**. Detailed change history for concept GO:0003700 ("sequence-specific DNA binding transcription factor activity") using the concept-based analysis module of OnEX.

OnEX is useful to find out which changes affected our application scenario. When inspecting the change history of GO:0003700 (Figure 6), we observe several attribute value changes of the concept name and description. Initially the concept was named "*transcription factor*", later "*transcription factor activity*" and currently it is denoted as "*sequence-specific DNA binding transcription factor activity*". Furthermore, its is_a relationships to parent concepts were revised. So in 2010-08, GO:0003700 was temporarily moved from parents GO:0030528 ("*transcription regulator activity*") and GO:0003677 ("*DNA binding*") to the MF root GO:0003674. In 2010-10, it was moved again to its new parent node GO:0001071 ("*nucleic acid binding transcription factor activity*"). The results in Figure 4(a) and 4(b) show that the former parent concept GO:0030528 was significant in the 2009-09 evaluation but no longer in 2011-03 due to the significant structural changes in this region. Figure 4(c) also reveals a reduced number of indirect (propagated) annotations for GO:0030528 in 2011-03 due to the lack of the incoming edges from the moved GO:0003700 concept (reducing the number of indirect annotations from ~1150 to only ~440). This underlines that term enrichment algorithms depend much on indirectly propagated annotations and therefore on structural ontology changes.

In addition to individual concept histories, we next analyze the stability of larger ontology regions using the Region Analyzer tool.

### Evolving Ontology Regions

The Region Analyzer of GOMMA [19] enables users to discover evolving and stable regions in large life science ontologies. This can be valuable to decide if there is a need to rerun ontology-dependent analysis applications like for functional profiling of large gene sets. The knowledge about strongly and marginal changing ontology regions may indicate that these regions are of special interest (unstable), have been neglected or are already complete (stable). As a manual discovery of such ontology regions is not feasible for large life science ontologies, automatic techniques can help to understand ontology evolution by providing (a helpful) assistance to ontology developers, curators and users.

**Figure 7:**
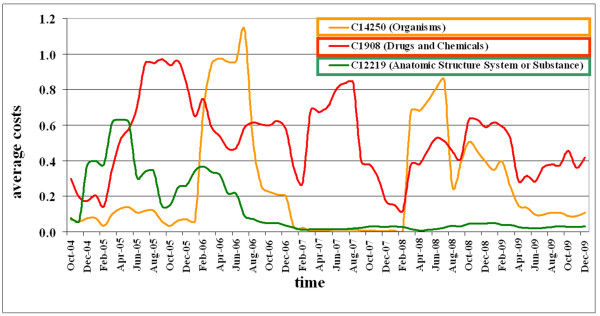
**Long-term region analysis for top-level concepts of NCI Thesaurus**. Tracking of average costs for sample regions in NCI Thesaurus between 2004 and 2009.

The region detection algorithm allows tracking the stability of ontology regions over time. Figure 7 displays the development of average change costs in NCI Thesaurus between 2004 and 2009 for three selected main categories. The computation used a sliding window of 'half year' size (window step: 1 month). This trend analysis exposed different evolution patterns. *"Drugs and Chemicals" *strongly evolved (red line) and were thus unstable over the whole period. Such regions represent very active research fields and may be modified in the future again. The *"Organism" *region (orange line) had periods of high and low stability. The periods of high instability may be influenced by new research findings or restructuring decision in the ontology consortium. Finally, the *"Anatomic structure or substance" *region (green line) remained more or less stable since 2007, indicating that the development of the anatomy part of the NCI Thesaurus may be almost finished as it covers accepted and standardized knowledge.

**Figure 8:**
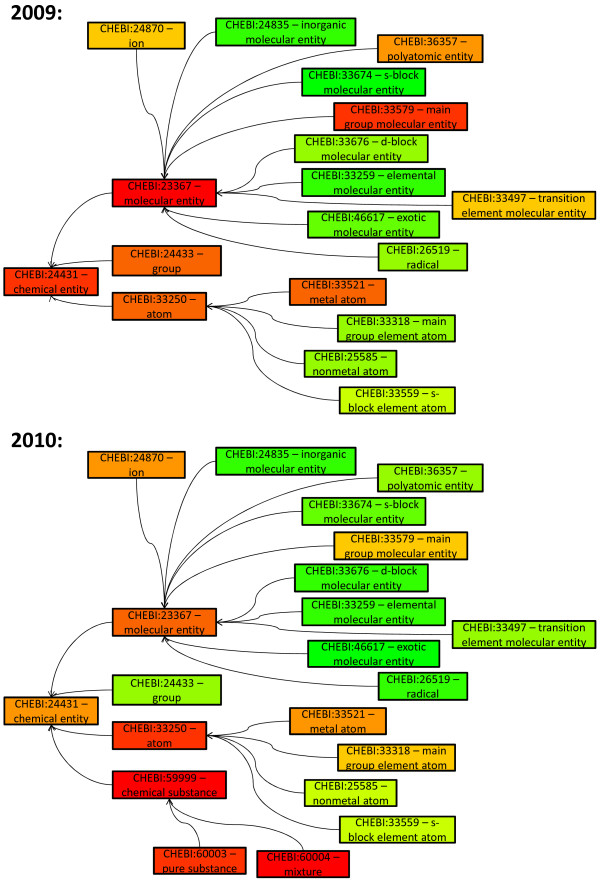
**Comparative region analysis for top-level concepts of ChEBI**. The figure shows the results of a region analysis for ChEBI top-level concepts. Red (green) categories evolved heavily (marginally) in the observation period and are thus unstable (stable). We analysed monthly released versions in 2009 (top) and 2010 (bottom).

Figure 8 shows the region stability for the top-level categories of ChEBI (Chemical Entities of Biological Interest) [48]. We distinguish two periods, particular we investigated all released versions in 2009 (top) and all releases in 2010 (bottom). The root (ChEBI:24431 - *chemical entity*) representing the overall change intensity shows an increased instability in both periods. However, there are differences for the other categories. For instance, in 2010 a new sub ontology on "*chemical substances" *(ChEBI:59999) with high instability has been introduced. On the one hand, there are regions possessing less changes in 2010 compared to 2009, e.g., *"group"*, *"polyatomic entity" *or *"transition element molecular entity"*. On the other hand, work in some regions has become more intensive, e.g., *"ion" *or *"group element atom"*.

**Figure 9:**
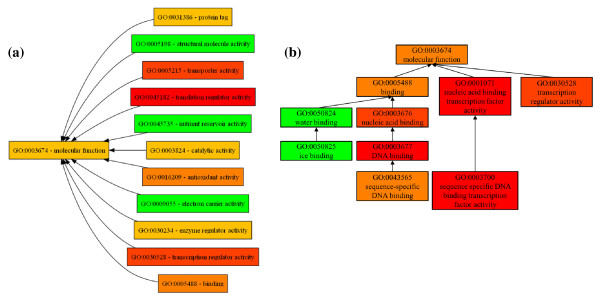
**Stable and unstable ontology regions in GO Molecular Functions using the Region Analyzer**. The figure shows the region stability of GO Molecular functions concepts between 2009-09 and 2011-03 (monthly versions). Red (green) categories evolved heavily (marginally) in the observation period and are thus unstable (stable). (a) Region stability of slim terms on the first level of GO Molecular function. (b) Region stability of the detected significant result concepts and their parents (from our application scenario in Figure 4).

Figure 9(a) exemplarily shows the region stability for slim terms (see http://www.geneontology.org/GO.slims.shtml) on the first level of GO-MF. Some of the top level slim terms remained completely stable, e.g., "*nutrient reservoir activity*" (GO:0045735) while others changed substantially, e.g. "*translation regulator activity*" (GO:0045182). Moreover, Figure 9(b) shows the region stability for the significant categories in our example scenario in the period 2009-09 to 2011-03 (monthly versions). Two concepts are completely stable (green), three show intermediate stability and four concepts are unstable. Especially the region of the newly introduced concept GO:0001071 and its child GO:0003700 are unstable. Interestingly, concepts *"water binding" *(GO:0050824) and *"ice binding" *(GO:0050825) remained stable and still appear only in the 2011-03 result set. This could be indirectly caused, e.g., the number of the annotated genes (one important input of the used hypergeometric test) decreased due to information reducing operations, such as setting concepts to obsolete or concept merges.

### COntoDiff

**Figure 10:**
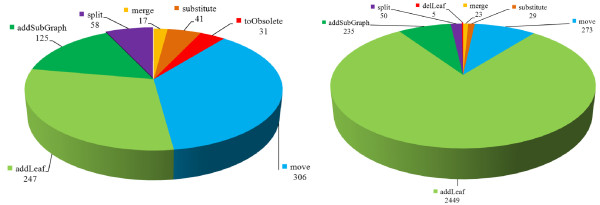
**Complex change operations in Mammalian Phenotype Ontology (left) and ChEBI (right)**. The diff for both ontologies was computed between the versions 2009-12 and 2010-12.

A further GOMMA tool is COntoDiff (Complex Ontology Diff) [18] which allows users to find complex changes between ontology versions such as merges or splits of concepts. In contrast to many simple add or delete changes, such complex changes are more meaningful and allow users to better understand how ontologies have changed. COntoDiff uses the rule-based change detection mechanism of GOMMA's DIFF component. For illustration, Figure 10 shows the number of found complex changes between versions of the MammalianPhenotype ontology (MP) as well as ChEBI between 2009-12 and 2010-12. First, there is a high number of information extending operations such as addLeaf, split as well as a significant amount of subgraph additions in both ontologies. This corresponds to the growth rates already shown in Table 3. In ChEBI addLeaf is the dominating change operation (a factor of 10 more addLeaf changes compared to MP). The subgraph additions provide information about what topics have been newly introduced. For instance, in MP a large subgraph "*increased tumor incidence*" (MP:0010274) was added between 2009-12 and 2010-12 and comprises 25 new concepts. The subgraph contains information about specific tumor incidences such as increased muscle or eye tumor incidence. In ChEBI the largest added subgraph *"organophosphate oxoanion" *(ChEBI:58945) contained 341 concepts. It covers organic phosphoric acid derivative in which one or more oxygen atoms of the phosphate group(s) has been deprotonated. However, there is also a significant amount of other complex changes such as concept merges or moves of concepts. In MP the operation merge([MP:0000442, MP:0008525], MP:0008525) fuses the concepts *"longitudinally short skull" *into *"decreased cranium height"*. In ChEBI "Ogawa trisaccharide 1" and "Ogawa trisaccharide 2" have been merged into CHEBI:52982. No concepts have been deleted in MP since it merely marks concepts as obsolete if they are no longer required or out-dated. In contrast five deletions of leaf concepts took place in ChEBI.

**Figure 11:**
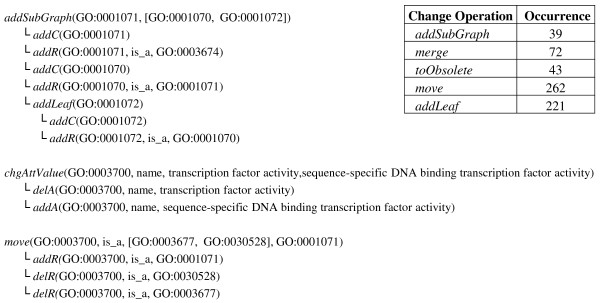
**Complex change operations in GO Molecular Functions**. The Figure shows three complex change operations that occurred in the region of the significant categories from our application scenario. The diff was computed between GO MF versions 2009-09 and 2011-03.

Finally, we analyzed how many complex changes affected GO-MF used in our application scenario. In GO-MF, there were 39 additions of subgraphs, 72 concept merges and 262 moves of concepts during our observation period (Figure 11). Our result set was especially affected by an addSubGraph operation with root concept GO:0001071 and the already mentioned move of concept GO:0003700 under its new parent concept GO:0001071.

The determined complex changes can be valuable in different application scenarios. For instance, they can be used for annotation migration similarly as discussed for the OnEX system. Furthermore, ontology-dependent applications and artifacts, e.g., queries or analysis algorithms (like our example scenario) can incorporate the changes. For example, queries referring to changed concepts could be adapted to work with the new ontology version.

## Discussion

We first compare GOMMA with other platforms and systems providing ontology management facilities. We then discuss some of the "Lessons Learned" from establishing and using the GOMMA infrastructure and its components. We finally present possibilities for Future Work.

### Comparison with other Platforms and Systems

Table 1 shows a selected set of existing platforms and systems for managing life science ontologies. There are several centralized hosting platforms, such as BioPortal [9] and Ontology Lookup Service (OLS) [8], that collect and provide search, navigation and download access to the most important life science ontologies. GOMMA provides similar functionalities with its Repository API, which is used by its tools such as OnEX to access and evaluate different ontology versions. Most platforms either are limited to the latest version of ontologies or only provide download access to older ontology versions without explicit information about the evolution. For instance, the Open Biomedical Ontology (OBO) Foundry [7] provides older ontology versions in the standardized OBO format. However, the versions can only be retrieved as compressed files from the OBO repository that is organized as a publicly accessible directory. By contrast, GOMMA supports an efficient, database-backed versioning of ontologies and provides a complex diff between ontology versions, i.e., users are able to recognize changes between released ontology versions. BioPortal and OBO offer pre-computed ontology mappings but do not take the occurred evolution of used ontologies into account. Thus, they face the problem that provided ontology mappings can become obsolete over time.

As surveyed in [23-28], many approaches have been proposed in the past to compute ontology mappings. For example, the SAMBO system [49] focuses on aligning and merging ontologies in the life sciences. It computes the alignments by using metadata, external knowledge (e.g., thesauri or documents) and learning techniques. GOMMA also provides metadata-based matching but supports different kinds of annotation-based matching using biomedical annotations. Furthermore, it provides a distributed architecture to enable an efficient parallel matching of large life science ontologies. GOMMA not only maintains multiple versions of ontologies but also multiple versions of ontology mappings. Matching of newer ontology versions can reuse older mappings and consider the stability of correspondences in the presence of changing ontologies. There are further approaches, such as provided in [50,51] aiming at enriching the GO by adding missing relationships between their sub-ontologies (functions, processes, and components). While in [50] metadata and annotation-based match approaches are combined, the approach introduced in [51] utilizes logic-based reasoners to derive the additional knowledge and to make existing ontologies consistent.

Tools such as Protégé [52] and KAON [53] support the user for ontology evolution. As a part of Protégé, the PromptDiff algorithm [54] allows for the computation of a structural diff using heuristic matchers. Changes such as add, delete or split are represented in a difference table. Moreover, in the life science domain there are several tools including OBOEdit [55] and OBO Explorer [56] to edit ontologies. By contrast, we focus on the management of (existing) ontology versions, difference computation as well as ontology evolution analyses.

Like OnEX [17], studies [57] and [58] aim at analyzing the evolution of biomedical ontologies. The presented method in [57] provides a colored graph visualization to help users recognizing added, deleted and changed concepts and relationships between two Gene Ontology versions. By contrast, OnEX provides tables and plots to quantitatively illustrate occurred ontology changes. It also allows interactive browsing of the ontology graph structure instead of showing a rather static graph picture [58] provides a simpler quantitative analysis of ontology changes than GOMMA (see [41]) and only considers concept and relationship frequencies as well as the maximal and the average path length in an ontology. Both [57] and [58] show results for the Gene Ontology only whereas [41] and OnEX are broader evolution studies including more than 750 versions of 16 life science ontologies.

In several of our previous work we focused on analyzing the evolution of life science ontologies and mappings. GOMMA is the infrastructure we used for most of these analyses and that unifies the previously published methods and techniques in a central system. As described in [20] GOMMA is able to manage various versions of mappings between two ontologies. That makes it possible to study the stability of mappings in detail. This feature is not provided by OnEX which focuses on exploring changes purely on the ontological level. Furthermore, with the help of the Match component one can use/combine the different match approaches (e.g., parallel matching [29], mapping composition [39]) in a common way. This is especially important when one has to deal with large life science ontologies. Finally the tools provided by GOMMA together may help users to better understand changes in and the evolution of ontologies as we have shown by studying the causes of changes in the term enrichment scenario.

### Lessons Learned

#### Scalability of the infrastructure to manage and analyze large data sets

Life science ontologies and corresponding mappings are usually very large ranging from several hundreds to thousands of concepts and correspondences. Versioning provides a further (time) dimension leading to increasing storage and processing requirements. The GOMMA versioning model avoids storing unchanged parts of a source between succeeding versions. The savings in storage requirements grow with the number of versions to be managed in the repository. At the same time, the approach has acceptable performance; typical ontology queries have an execution time lower than one second. The large data volumes also affect applicability and execution time of algorithms analyzing the data. In particular, the approaches for matching large ontologies are very memory- and computing-intensive. The distributed service-based GOMMA infrastructure and support for parallel matching proved to be effective for efficient ontology matching. A next possible step could be transferring the infrastructure to larger cloud environments to further increase scalability.

#### Generic data management

GOMMA's generic data management approach based on an attribute-value concept proved to be effective to uniformly manage heterogeneous ontologies, entity sources and mappings in the repository. Furthermore, the life time-based versioning concept of GOMMA could be uniformly utilized for ontology concepts and attributes, entity sources as well as mappings. Hence, the GOMMA functionality to determine ontology and evolution mappings and for evolution analysis can be utilized for a large spectrum of life science ontologies and entity sources.

#### Mappings as a key technology for algorithm development and analysis

The different kinds of mappings (annotation mappings, ontology mappings and evolution mappings) proved to be of key importance for the development of new algorithms and the evolution analysis of ontologies and annotations. Annotation mappings provided in different data sources could be utilized for annotation-based ontology matching as well as the stability analysis of ontologies. Ontology mappings determined by the GOMMA Match component are used by the DIFF component to identify complex changes between ontology versions. Another important use case for ontology mappings is the merge of multiple ontologies into one global ontology [16,59], e.g., the integration of multiple anatomical ontologies. Finally, evolution mappings summarize the change history of ontologies and help ontology curators and users to better deal with the effects of evolving ontologies, e.g., for migrating affected annotations. This holds particularly for evolution mappings consisting of complex changes typically modifying multiple ontology concepts and relationships. Such complex changes also make ontology evolution more understandable especially for large life science ontologies.

### Future Work

We plan to exploit the GOMMA infrastructure in further applications and make them available online for the life science community. Currently, we are working on a web service interface making the managed versions of ontologies, entity sources and mappings programmatically accessible for other applications. A new web application is planned to support interactive use of our approach of detecting stable and changing ontology regions. Finally, we are using the established infrastructure to analyze the impact of ontology and annotation evolution on application results, such as for gene enrichment analysis and ontology matching.

## Conclusions

We have presented GOMMA, a generic infrastructure for managing and analyzing life science ontologies and their evolution. The component-based infrastructure utilizes a generic repository to uniformly manage many versions of heterogeneous ontologies, entity sources and mappings. The functional components aim at matching life science ontologies, detecting and analyzing evolutionary changes and patterns in these ontologies. The infrastructure is used in several online available applications. OnEX provides several quantitative difference statistics and allows annotation migration while the Region Analyzer assesses the robustness of ontology regions. The proposed infrastructure is not limited to life sciences but could also be applied in other domains and communities including the Semantic Web.

## Availability and requirements

• **Project name: **GOMMA (**G**eneric **O**ntology **M**atching and Mapping **Ma**nagement)

• **Project home page: **http://dbs.uni-leipzig.de/GOMMA

• **Operating systems: **Platform independent

• **Programming language: **Java

• **Other requirements: **Java 1.5 or higher, MySQL

## Competing interests

The authors declare that they have no competing interests.

## Authors' contributions

TK, AG and MH designed and implemented the components of the GOMMA infrastructure. TK was responsible for the overall design of GOMMA especially the repository API and the data management. AG contributed to the Match component including mapping management/parallel matching and performed the term enrichment application scenario. The ontology-specific parts including the tools OnEX and Region Analyzer were realized by MH. ER supervised and coordinated the project. All authors have contributed to, read and approved the final manuscript.
